# An investigation of the level of stigma and the factors influencing it in the rehabilitation of young and middle-aged stroke patients-a cross-sectional study

**DOI:** 10.1186/s12883-023-03189-4

**Published:** 2023-04-01

**Authors:** Zixiu Zheng, Runluo Song, Yunxiao Zhao, Hongxia Lv, Yanqing Wang, Cong Yu

**Affiliations:** 1 Inner Mongolia Baogang Hospital, Baotou, China; 2 Henan University of Science and Technology, Luoyang, China; 3 Department of Nursing, Shenzhen Second People’s Hospital, Shenzhen, China

**Keywords:** Young and middle-aged, Stroke, Stigma, Rehabilitation, Influencing factors

## Abstract

**Background:**

There are few reported studies on stigma in young and middle-aged stroke patients during the rehabilitation period, however, the rehabilitation period plays a key role in the patients’ disease regression. Exploring the level of stigma and the influencing factors in young and middle-aged stroke patients during the rehabilitation period is crucial for determining how to reduce the level of stigma and improve the patients’ motivation for rehabilitation treatment. Therefore, this study investigated the level of stigma in young and middle-aged stroke patients and analyzed the factors influencing stigma in order to provide a reference or basis for healthcare professionals to develop effective and targeted stigma intervention programs.

**Methods:**

Using a convenience sampling method, 285 young and middle-aged stroke patients admitted to the rehabilitation medicine department of a tertiary care hospital in Shenzhen, China, from November 2021 to September 2022 were selected and surveyed using a general information questionnaire, the Stroke Stigma Scale(SSS), the Barthel Index(BI), and the Positive and Negative Emotions Scale(PANAS), and multiple linear regression and smoothed curve fitting were used to analyze the factors influencing the stigma of young and middle-aged stroke patients during the rehabilitation period.

**Results:**

SSS score of 45.08 ± 11.06, univariate analysis of age, occupation, education level, pre-stroke monthly income, insurance type, comorbid chronic disease status, primary caregiver, BI, positive and negative emotion as factors influencing stigma. Multiple linear regression showed that age, pre-stroke monthly income, BI, positive and negative emotions were independent influences on stigma in young and middle-aged stroke patients, explaining 58.0% of the total variance in stigma. A smoothed curve fit revealed a curvilinear relationship between the above influences and stigma.

**Conclusion:**

Young and middle-aged stroke patients have a moderate level of stigma. Medical staff should focus on young patients aged 18–44 years, those with high monthly income before the stroke, those with poor self-care ability, and those with low positive and high negative emotion scores, and conduct early assessments and adopt targeted intervention programs according to the influencing factors to reduce the stigma of young and middle-aged stroke patients, improve their motivation for rehabilitation, and help them return to their families and society as soon as possible.

**Trial Registration:**

Registration number of China Clinical Trials Registration Center: 20,220,328,004-FS01.

**Supplementary Information:**

The online version contains supplementary material available at 10.1186/s12883-023-03189-4.

## Introduction

Stroke is a group of acute episodes of neurological deficits caused by multiple causes of local cerebral blood circulation disorders, also known as stroke or cerebrovascular accident [1]. In recent years, the incidence of stroke has shown a trend toward youth [2, 3].

It is the second-leading cause of death and disability worldwide [4–6], with five major characteristics: high morbidity, disability, mortality, recurrence, and economic burden [7]. However, the problem of stroke is more serious and prominent in China, with a recent study showing that the overall prevalence, morbidity, and mortality of stroke among adults aged 40 years and older is estimated to be 2.6% in 2020 [8], and stroke has been classified as a major chronic noncommunicable disease in China and is the leading cause of death and disability among Chinese adults [7],Stroke has become a disaster, threatening human life, health, and quality of life [9].

Stigma, originally from the Greek word meaning “brand,“ was first introduced by the American sociologist Goffman in 1963 and refers to an internal experience of shame caused by discrimination and isolation from others because of a disease [10]. Stroke can lead to neurological deficits such as hemiplegia, aphasia, hemianesthesia, ataxia, cognitive impairment, and other neurological deficits that require social and family support to live [11]. As the mainstay of society, young and middle-aged people have important personal, family and social responsibilities, and are the best time to realize their self- worth and create wealth. After stroke, patients are prone to different degrees of stigma, such as sensitivity, low self-esteem, and frustration. Stigma leads to loneliness and social isolation [12], decreases motivation and initiative for rehabilitation treatment, hinders the process of rehabilitation [13], greatly affects patients’ physical and mental health and quality of life [14, 15], and brings a heavy burden to families and society.

Stigma is commonly present in stroke patients, but at inconsistent levels [16]. It has been reported in research that acute stroke in young people differs from acute stroke in non-young people in terms of risk factor distribution, stroke subtypes, stroke severity, and outcome [17]. However, most current studies on the stigma of stroke patients do not delineate characteristics such as differences in population and pathology [18]. In one study comparing stroke patients using 55 years of age as a cut-off, it was concluded that it is crucial to focus on the variability in age in different populations [17]. As a result, this study investigated the level of stigma in young and middle-aged stroke patients during recovery, using 45 years of age as the cut-off value, and examined the factors associated with stigma, with the goal of providing a reference or foundation for healthcare professionals to develop effective and targeted stigma intervention programs.

## Methods

### Research subjects

Young and middle-aged stroke patients hospitalized in the rehabilitation medicine department of a tertiary care hospital in Shenzhen, China, were selected as the study population using a convenience sampling method. Inclusion criteria: (1) meeting stroke diagnostic criteria [19]; (2) 18 years ≤ age < 65 years; (3) having clear consciousness and ability to correctly understand the content of the questionnaire. (4) informed consent is required to participate in this study. Exclusion criteria: (1) complicated with various major diseases; (2) patients who were participating in other studies.

### Research tools

#### General information questionnaire

It was designed by the investigators themselves and included two sections with demographic and sociological information (gender, age, marital status, occupation, education, pre-stroke monthly income, insurance type, smoking, drinking, primary caregiver) and disease-related information (comorbid chronic diseases, number of strokes, genetic history).

#### Stroke stigma scale (SSS)

The SSS, constructed by Zhu Minfang et al. [20]in 2018, is a dedicated scale to assess the stigma of stroke patients with 4 dimensions and 16 entries, with the 4 dimensions being self-perception, somatic impairment, discrimination experience, and social interaction. The scale has a total score of 16–80. The higher the total score, the higher the degree of stigmatization. The Cronbach’s alpha for the total scale was 0.916, the retest reliability was 0.924, and the CVI was 0.89 [21], and the total scale score was divided into five states: very high, high, moderate, low, and low [22].

#### Positive and negative affect scale (PANAS)

The scale was developed by Watson et al. [23] In 1988, it consists of 20 items, 10 items each for positive and negative emotions, and is rated on a 5-point Likert scale, with “almost none” scoring 1, “relatively little” scoring 2, “moderately” scoring 3, “more” scoring 4, and “and more” scoring 5. The Cronbach’s alpha for positive and negative emotions were 0.937 and 0.919, respectively [24].

#### Barthel index (BI)

It is an international rehabilitation medicine method for assessing patients’ ability to perform activities of daily living [25], designed by Mahoney and Barthel [26], with 10 items that rate patients’ self-care in eating, washing, grooming, dressing, bowel control, urination, toileting, bed and wheelchair transfer, level walking, and stair walking. The higher the score, the greater the independence; the ability to perform activities of daily living was independent between a total score of 80–100, a total score of 61–79 needing help, a total score of 40–60 partially dependent, a total score of 20–39 very dependent, and a total score of less than 20 completely dependent [27], with a Cronbach’s alpha of 0.90 [28].

### Information collection

The researcher was mainly responsible for the receipt and distribution of questionnaires, and another graduate student assisted. Prior to the survey, the unified instructional language was used to explain the purpose of the survey and the method of filling out the questionnaire to the respondents, etc. All questionnaires were anonymous and collected on the spot after completion, and any omissions were made up in time.

### Statistical methods

*SPSS25.0* Statistical software was used to analyze the data, and the count data were expressed as cases and the measurement data were expressed as mean ± standard deviation. *t*-test, one-way ANOVA, or rank sum test were used for comparison between groups; *Pearson* Correlation analysis was used to explore the correlation between sickness stigma and the Barthel index, positive emotion, and negative emotion; multiple linear regression was used to Multiple linear regression was used to analyze the factors influencing the sense of stigma; statistical packages *R* language and *Empower Stats* were used for smoothing curve-fitting analysis, and all statistical analyses were performed with *P*<0.05, indicating statistically significant differences.

## Results

### Description of the patient screening process

**Fig. 1:**
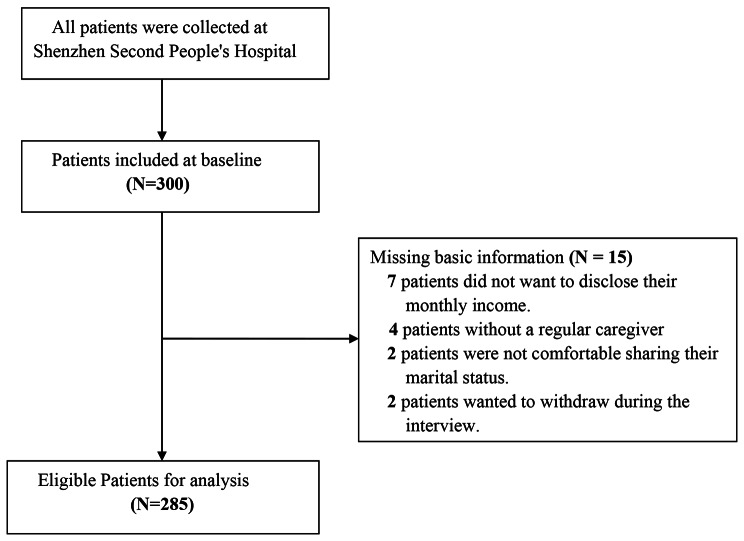
Description of the participant screening process

In this study, 300 patients were initially included, and 15 cases with missing basic information were excluded, leaving 285 cases for the final data analysis, as detailed in the flow chart (Fig. 1).

### General data description of young and middle-aged stroke patients

The results showed statistically significant differences (*P* <0.05) by age, occupation, education level, monthly income, insurance type, comorbid chronic disease, and primary caregiver, as shown in Table 1.

**Table 1:** Description of general data of young and middle-aged stroke patients (N = 285)

Projects	Classification	Number of cases (n)	SSS	Test value	*P*
Gender	Male	211	45.22 ± 11.62	0.362^a^	0.717
	Female	74	44.68 ± 9.34		
Age(years)	18–44	39	51.38 ± 13.01	3.342^a^	**0.002**
	45–64	246	44.08 ± 10.40		
Marital Status	Married	277	44.88 ± 11.10	1.069^b^	0.363
	Unmarried	6	52.50 ± 8.41		
	Widowed	1	49.00 ± 0.00		
	Divorced	1	51.00 ± 0.00		
Occupation	Employee	214	46.11 ± 11.35	4.300^b^	**0.005**
	Businessmen	21	43.48 ± 10.45		
	Farmer	25	38.04 ± 9.20		
	Unemployed	25	44.64 ± 8.02		
Education	Primary	28	40.68 ± 9.48	13.825^c^	**0.008**
	middle school	85	45.15 ± 9.83		
	High School	104	43.82 ± 11.78		
	University	66	48.55 ± 11.11		
	Graduate	2	54.50 ± 13.44		
Monthly Income (¥)	<1000	41	41.88 ± 8.29	14.459^c^	**0.006**
	1000–3000	24	42.25 ± 9.26		
	3000–5000	79	43.33 ± 10.91		
	5000–10,000	97	46.46 ± 11.63		
	>10,000	44	49.68 ± 11.57		
Insurance type	Urban Insurance	227	46.08 ± 11.25	3.742^b^	**0.012**
	Resident Insurance	15	44.40 ± 9.21		
	Rural Insurance	36	40.08 ± 9.47		
	Self-funded	7	39.71 ± 8.79		
Smoking	No smoking	159	45.35 ± 10.75	0.460^a^	0.646
	Smoking	126	44.74 ± 11.47		
Drinking	No drinking	172	44.97 ± 11.05	0.211^a^	0.833
	Drinking	113	45.25 ± 11.11		
Comorbid chronic disease	None	70	44.06 ± 12.25	4.776^b^	**0.003**
	Hypertension	161	46.17 ± 10.05		
	Diabetes	20	36.95 ± 9.62		
	Hypertension and Diabetes	34	46.76 ± 12.00		
Number of strokes	Primary	229	45.52 ± 11.18	2.444^c^	0.486
	Secondary	50	43.00 ± 10.63		
	Tertiary	5	44.20 ± 9.42		
	Fourth	1	53.00 ± 0.00		
Primary Caregiver	Spouse	123	45.59 ± 11.61	11.313^b^	**0.000**
	Children	42	41.93 ± 10.02		
	Relatives	28	52.36 ± 11.06		
	Caregiver	54	48.17 ± 9.23		
	Self-care	38	37.16 ± 6.41		
Genetic history	No	127	44.69 ± 10.08	0.536^a^	0.592
	Yes	158	45.39 ± 11.81		

Note: a represents *t-test*; b represents *F*-test; c represents *H*-test

### SSS, PANAS and BI scores of young and middle-aged stroke patients

The results of the study showed that the SSS score for young and middle-aged people are 45.08 ± 11.06, and the mean scores of the entries for each dimension of the scale were, in descending order, 3.18 ± 1.21 for social interaction, 3.14 ± 0.942 for self-perception, 3.03 ± 0.913 for somatic disorders, 2.20 ± 0.67 for experiences of discrimination, and PANAS and BI scores, as shown in Table 2.

**Table 2:** Scores on each scale (N = 285)

Projects	Grouping	Entry	Score	Average score of entries
SSS	Somatic Disorders	4	12.18 + 3.47	3.03 + 0.91
	Social Interaction	3	9.69 + 3.64	3.18 + 1.21
	Experience of discrimination	4	8.34 + 2.66	2.20 + 0.67
	Self-perception	5	14.87 + 4.71	3.14 + 0.94
	Total sickness stigma score	16	45.08 + 11.06	2.82 + 0.70
PANAS	Positive emotions	10	20.20 + 6.20	2.02 + 0.62
	Negative emotions	10	22.68 + 7.16	2.27 + 0.72
BI	< 20 points	-	53.33 + 11.02	-
	20–39 points	-	50.43 + 6.53	-
	40–60 points	-	49.2 + 11.17	-
	61–79 points	-	46.75 + 9.32	-
	80–100 points	-	40.19 + 10.37	-

### Description of the linear relationship of factors influencing disease stigma in young and middle-aged stroke patients

#### Correlation analysis of SSS with the BI and positive and negative emotion scales

*Pearson* correlation analysis showed that SSS is negatively correlated with the BI, negatively correlated with positive emotions, and positively correlated with negative emotions, with statistically significant differences (*P* < 0.01), as shown in Table 3.

**Table 3:** Correlation of SSS with and BI and PANAS (N = 285)

Projects	SSS	BI	Positive emotions	Negative emotions
SSS	1			
BI	− 0.391**	1		
Positive emotions	− 0.595**	0.497**	1	
Negative emotions	0.687**	− 0.387**	− 0.620**	1

** Significant correlation at the 0.01 level (two-tailed).

#### Multiple linear regression analysis of the factors influencing stigma

The SSS for young and middle-aged stroke patients was used as the dependent variable, and statistically significant data (age, occupation, education, monthly income, type of health insurance, chronic disease status, primary caregiver), the Barthel Index, positive emotions, and negative emotions were used as independent variables in a multiple linear regression analysis. The independent variables were assigned as shown in Table 4. The linear regression results showed that age, monthly income, the BI, positive emotions, and negative emotions were independent influences on the level of stigma (*P* < 0.05), and the model was well fitted (*R*^*2*^of 0.580), meaning that the variables together explained 58.0% of the total variation in stigma in young and middle-aged stroke patients, as shown in Table 5.

**Table 4:** Assignment of independent variables

Projects	Assignment Description
Age (years)	18–44 years = 1, 45–64 years = 2
Occupation	Employee = 1, Businessman = 2, Farmer = 3, Unemployed = 4
Education	Primary = 1, middle school = 2, high school = 3, University = 4, Graduate = 5
Monthly income (**¥**)	<1000 = 1, 1000–2999 = 2, 3000–4999 = 3, 5000–10,000 = 4, >10,000 = 5
Insurance type	Urban Insurance = 1, Resident Insurance = 2, Rural Insurance = 3, Self-funded = 4
Comorbid chronic diseases	None = 0, hypertension = 1, diabetes = 2, hypertension and diabetes = 3
Primary caregiver	Spouse = 1, Children = 2, Caregiver = 3, Self-care = 4
BI	Original Value Carried
Positive emotions	Original Value Carried
Negative emotions	Original Value Carried

**Table 5:** Multiple linear regression analysis of factors influencing stigma (N = 285)

Independent variable	Unstandardized coefficient		*Beta*	*t*	*P*	*VIF*
	*B*	Standard error				
(Constant)	74.03	5.771		12.827	0.000	
Age	-0.351	0.071	-0.256	-4.95	**0.000**	1.270
Occupation	0.796	0.776	0.070	1.025	0.306	2.236
Education	0.152	0.712	0.013	0.214	0.831	1.770
Monthly income	1.589	0.648	0.179	2.451	**0.015**	2.525
Insurance type	-1.471	0.826	-0.107	-1.781	0.076	1.726
Comorbid chronic diseases	0.606	0.581	0.049	1.043	0.298	1.054
Primary caregiver	-0.509	0.342	-0.070	-1.487	0.138	1.057
BI	-2.221	0.473	-0.237	-4.696	**0.000**	1.216
Positive emotions	-0.422	0.095	-0.248	-4.460	**0.000**	1.878
Negative emotions	0.684	0.079	0.440	8.614	**0.000**	1.720
*R*^*2*^ = 0.595, Adjust *R*^*2*^ = 0.580						
* F* = 23.088						
*P*<0.001						
Dependent variable: SSS						

### Description of the non-linear relationship of factors influencing stigma in young and middle-aged stroke patients

#### Relationship between general information and stigma

**Fig. 2:**
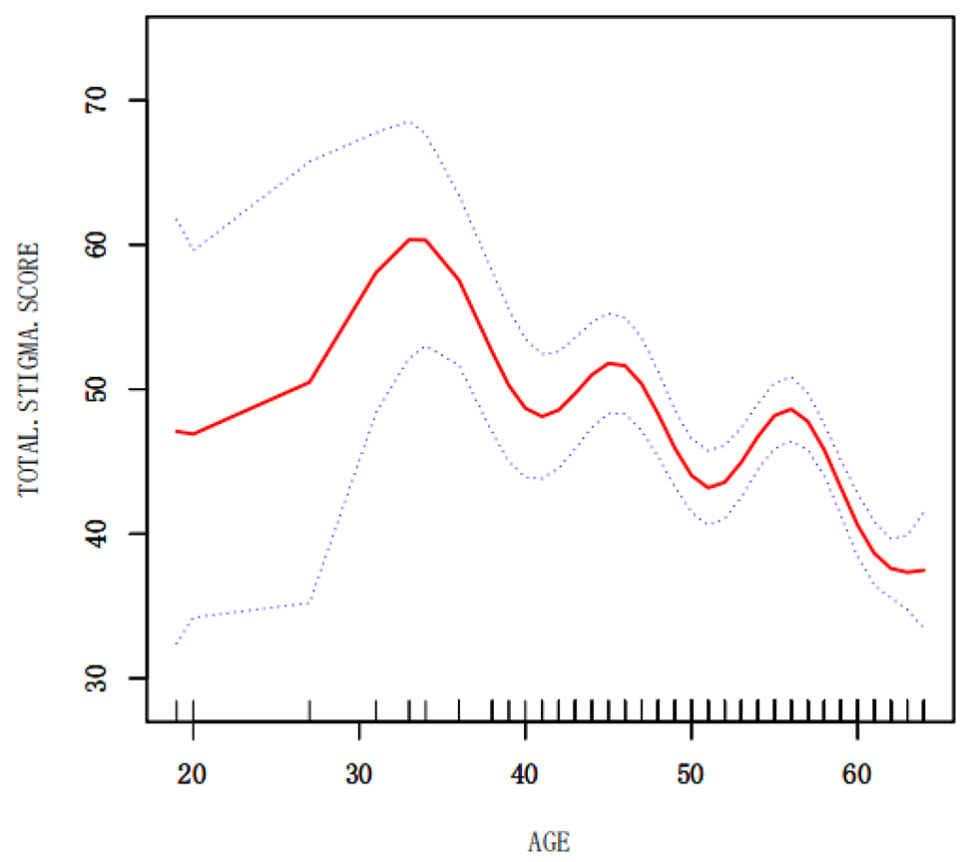
The relationship between age and stigma

**Fig. 3:**
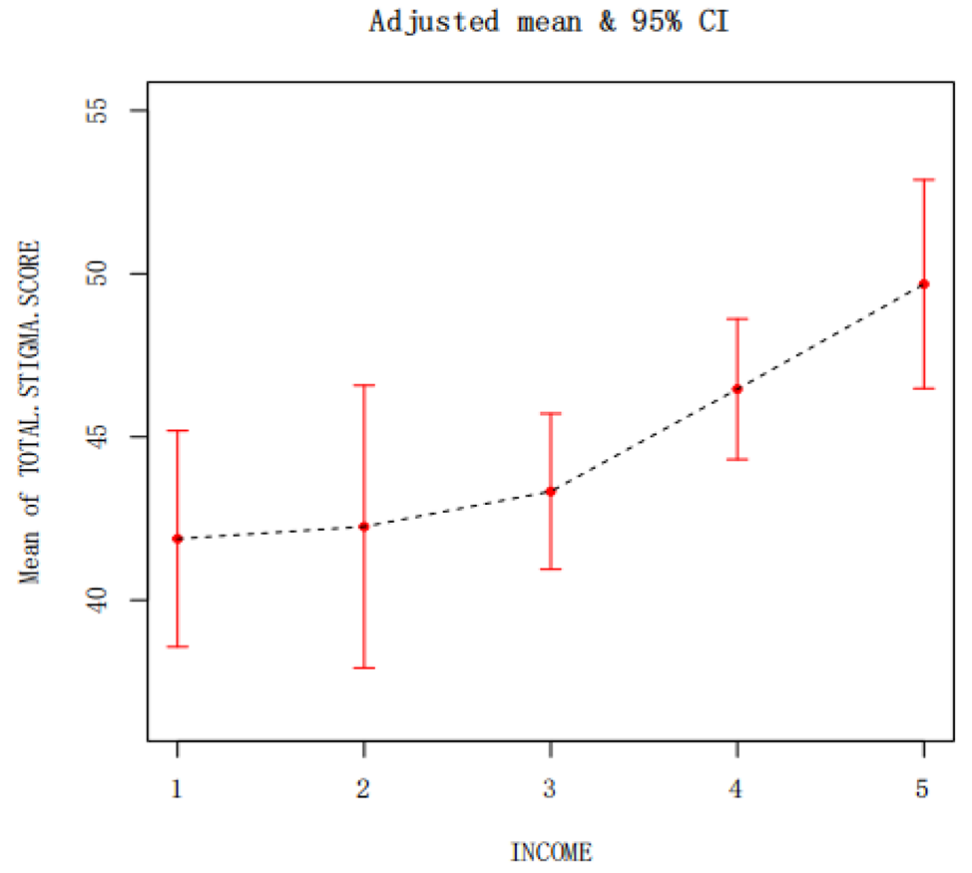
The relationship between income and stigma

The general information (age and monthly income) that was significant in the multiple linear regression analysis was described by smoothing curve fitting, where the red solid line indicates the smoothed curve fitting between the variables and the blue dashed line indicates the 95% confidence interval of the fitting. A non-linear relationship was observed between age and stigma, with different trends in stigma levels among patients of different ages but a general trend of decreasing stigma levels with increasing age (Fig. 2). Using monthly income as a categorical variable (Table 4), a smoothed curve fit allowed us to observe a more pronounced increase in the level of stigma for patients with a monthly income greater than ¥5,000 (Fig. 3).

#### The relationship between BI and stigma

**Fig. 4:**
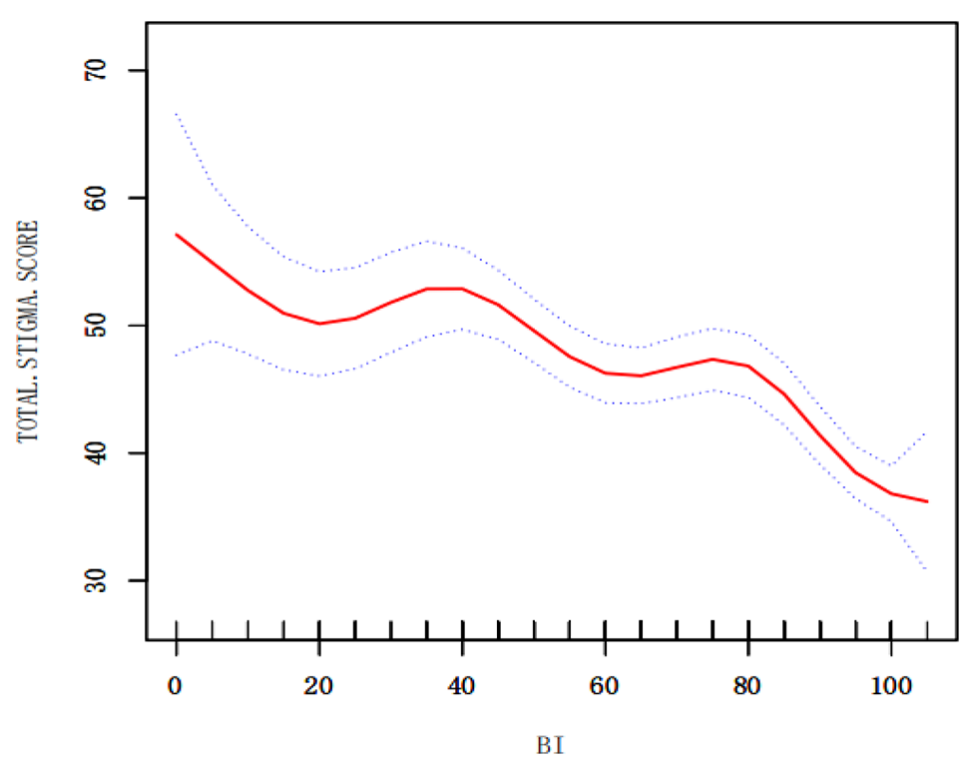
The relationship between BI and stigma

The non-linear relationship between BI and stigma was observed by smoothed curve fitting, where the red solid line indicates the smoothed curve fitting between the variables and the blue dashed line indicates the 95% confidence interval of the fitting, where it can be observed that there are different decreasing trends of the stigma curve for patients with different independent levels of daily living self-care (Fig. 4).

#### Relationship between PANAS and stigma

**Fig. 5:**
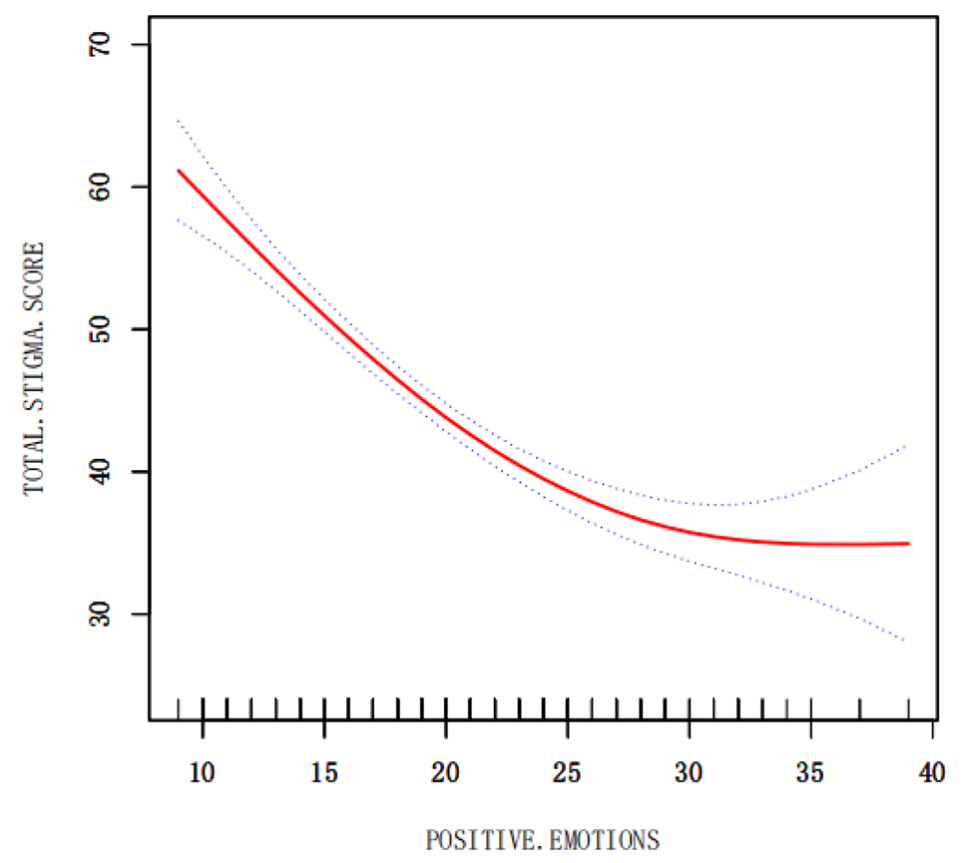
The relationship between positive emotion and stigma

**Fig. 6:**
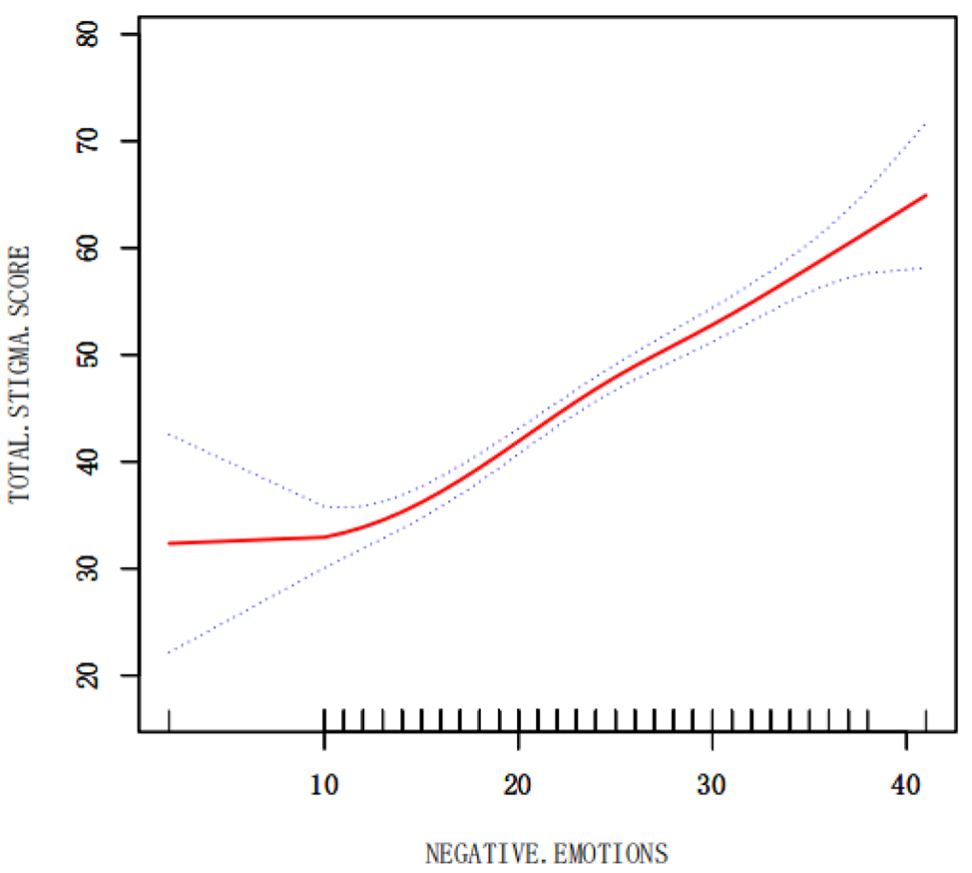
The relationship between negative emotion and stigma

The relationship between positive and negative emotions and stigma was observed through a smoothed curve fit, where the red solid line indicates the smoothed curve fit between the variables and the blue dashed line indicates the 95% confidence interval of the fit. We observed that when positive emotions were scored at 30, the total score of stigma would not decrease (Fig. 5), while negative emotions, when scored above 10, increased significantly with the increase of negative emotions (Fig. 6).

## Discussion

### Current status of stigma in young and middle-aged patients recovering from stroke

The results of this study showed that the total stigma score of 45.08 ± 11.06 in young and middle-aged patients recovering from stroke is at an intermediate level according to the Stroke Stigma Scale, which is consistent with SARFO [29] 2017 study in West Africa on the status of 200 stroke patients, but higher than the results of Li Mulin [30]. The reason for this is that the sample of this study was young and middle-aged people, and stroke emergencies may be more physically and mentally devastating to young and middle-aged people. Combined with the findings in Table 1, the highest score of 3.18 ± 1.21 was obtained for the social interaction entry among the four dimensions of the SSS, indicating that stroke has a serious impact on patients’ social interaction, probably because patients consider themselves a burden to others after stroke and their social circle becomes smaller, bordering on no social interaction. Therefore, patients’ needs should be understood in time, and regular patient communication activities should be conducted according to the patients’ needs to promote harmonious interpersonal relationships, meet the patients’ social needs, and reduce the patients’ level of stigma. The second highest score was for self-perception, with 3.14 ± 0.94, indicating that patients’ sense of shame comes mainly from their own feelings, and that stroke is a heavy psychological blow to the patients themselves, who may feel humiliated and less valued and respected than before. Therefore, psychological interventions for young and middle-aged stroke patients are essential to reduce the intrinsic stigma of the disease. The somatic disorder entry scored third. Stroke causes neurological symptoms such as hemiplegia, aphasia, hemianesthesia, ataxia, swallowing disorder, etc. Patients feel sad that they cannot do some things and feel uncomfortable with the way others assist them, and the slow and costly recovery process makes them prone to stigma. However, the lowest score of 2.20 ± 0.67 was obtained for the dimension of experience with discrimination, indicating that society accepts stroke patients well and does not treat them differently or discriminate against them. Therefore, health care professionals should use the scores of each dimension of the SSS as a reference to actively explore scientific and effective intervention methods to reduce the stigma of young and middle-aged stroke patients in the recovery period.

### Factors influencing stigma in young and middle-aged patients recovering from stroke

#### Age was negatively correlated with the level of stigma

According to the criteria for delineating the age of Chinese residents and the age cut-off for Chinese residents in previous studies in the cardiovascular literature, middle-aged and young adults are defined as 18–64 years old, 18–44 years old as young adults, and 45–64 years old as middle-aged [31]. The results of this study showed that when age was used as a categorical variable, young patients aged 18–44 years with stroke recovery had a total stigma score of 51.38 ± 13.01, which was a high level of stigma, and middle-aged patients aged 45–64 years had a total stigma score of 44.08 ± 10.40, which was at an intermediate level, showing that the level of stigma was more significant in young patients, in line with Yin Chunlan [32] in 2019 on 277 young breast cancer patients with similar findings. However, when age is used as a continuous variable, it can be found from Fig. 2 that age and stigma are not simply linear, and the trend of stigma level varies among patients of different ages, but the overall trend is that the level of stigma decreases with increasing age, and the reason for this is that, On the one hand, young stroke patients are on the rise in their studies, families, and careers, and a stroke event greatly affects work, family, and social life, whereas middle-aged patients have become more stable in these areas. On the other hand, about 75% of stroke patients have residual functional impairment [33]. In contrast, young stroke patients are more conscious of their image and may have a more pronounced sense of stigma. At present, it is found that there are literature studies on the sense of stigma of stroke patients, but there are few literature studies on young and middle-aged stroke patients in rehabilitation alone. This study shows that the problem of sense of stigma of young patients is more prominent. As a result, medical professionals should not ignore the stigma of young and middle-aged stroke patients during rehabilitation, especially in young stroke patients, and should assess and develop practical interventions to reduce stigma and increase motivation for rehabilitation as soon as possible.

#### Monthly income was positively correlated with the level of stigma

In 2021, the average annual salary of urban non-private sector employees in 31 provinces in China was ¥106,837 [34]. Shenzhen is at the forefront of China’s reform and opening up and the first special economic zone in China. The average annual salary was ¥153,471 [35], higher than the national level, and the same consumption level is higher than the national level. However, the functional reconstruction and recovery of stroke patients during their rehabilitation period is a relatively long process. It brings a huge economic burden to patients and their families and also poses challenges to the public health of society. The results of this study showed that the pre-stroke monthly income of young and middle-aged stroke patients was negatively correlated with stigma, with patients with a monthly income greater than ¥10,000 having a high level of stigma at 49.68 ± 11.57 and patients with a monthly income <¥1000 having a medium level of stigma at 41.88 ± 8.29. The higher the monthly income before the disease, the higher the level of stigma in the recovery period, and from Fig. 3, it can be seen that the level of stigma rises more significantly in patients with a monthly income greater than ¥5000, which is inconsistent with the findings of Wu [36] in their survey of 260 stroke patients in 2019, where Wu concluded that patients with a monthly income of less than ¥3000 had a more significant sense of illness shame. The analysis of the different results may be due to the following reasons: First, Wu’s study did not consider the age of the population, whereas the present study included young and middle-aged stroke patients, and young and middle-aged patients with high monthly income had a more affluent material life before the disease. The psychological gap between patients’ high income before the disease and no income after the disease is large, which can easily lead to low self-esteem and shame. Secondly, patients with high monthly incomes have a relatively high social status and have been perceived as successful by their colleagues and family members, and the sudden loss of social function and social identity of the messenger after the disease leads to a more pronounced sense of shame. Finally, The subject of our study is the city of Shenzhen, a special economic zone in China, patients with high monthly incomes are the main breadwinners of their families, and after the disease, they not only lose their main source of income, but also cause financial burdens on their families and become indebted to their families, and are also prone to negative feelings such as shame, low self-esteem, and guilt, which lead to high levels of shame. Therefore, health care workers should pay attention to young and middle-aged stroke patients with high income before the disease and provide early targeted interventions to reduce the patients’ stigma, so that they can actively cooperate with rehabilitation treatment and return to society as soon as possible.

#### BI was negatively correlated with the level of stigma

This study found that the poorer the ability to perform daily living, the higher the stigma score, which is consistent with a cross-sectional survey of 72 stroke patients by Tong Qi [37] in 2020 and with Anderson [38] and Silva [39]studies. In the results of this study, young and middle-aged stroke patients had a Barthel Index score of 69.07 ± 26.04, indicating that they needed partial assistance in activities of daily living and could not perform them independently. From Fig. 4, it can be seen that there are different decreasing trends for different stigma curves for patients’ levels of independence in daily life care. The analysis of possible reasons for this is as follows: On the one hand, on the personal side, patients believe that they are young and well and that stroke is irrelevant or far away from them, so the limitation of daily life activities after stroke brings a heavy blow to patients and easily leads to stigma. On the other hand, in terms of family responsibilities, this group of young and middle-aged people is responsible for taking care of the elderly and children. After the disease, they not only cannot fulfill their family responsibilities, but also need the care and help of their family members and drag them down, which easily generates guilt, self-blame, and shame, coupled with the fact that rehabilitation is a longer process and the recovery of functional activities is not obvious. Therefore, during the rehabilitation phase of young and middle-aged stroke patients, medical and nursing staff take timely and effective measures according to the patients’ ability to perform activities of daily living and provide psychological guidance to reduce the patients’ sense of shame.

#### Emotional state correlates with disease stigma

The present study showed that positive emotions were negatively correlated with stigma and negative emotions were positively correlated with stigma in young and middle-aged patients recovering from stroke, which is In line with Wang Xiao [40], emotional state was closely related to patients’ motivation for treatment [41]. The positive emotion score in this study was 20.20 ± 6.19, and the negative emotion score was 22.68 ± 7.16. The positive emotion score was low, and during the researcher’s questionnaire collection, some patients expressed that their rehabilitation progress was slow and that there was a big gap between their expected rehabilitation effects. Some patients were not willing to communicate with the rehabilitation therapist because they could not speak clearly, and they were only mechanically accomplishing their tasks in the rehabilitation exercise. Li Shichen [42]After a 5-year follow-up of 277 breast cancer patients, it was found that positive emotions had a more significant and long-lasting effect on patients’ prognosis than negative emotions, and it was also observed in Fig. 5 that when positive emotions reached 30 points, the level of stigma was lower and at a stable level. Therefore, it is urgent to advocate for more studies focusing on the positive emotions of patients, and to explore and enhance the positive emotions of patients, so as to reduce the stigma of patients and improve the motivation of patients in rehabilitation treatment.

However, there are some limitations to our study. On one side, it was a cross-sectional survey and did not follow up on the patients’ sense of stigma. On the other hand, because small vessel disease of the brain is a heterogeneous series of pathophysiological processes [43, 44], with assorted mechanisms and clinical outcomes, it is necessary to narrow the type of stroke population [45], Therefore, we recommend that future studies investigate and analyze the relationship and relevance of small vessel disease versus other ischemic subtypes at the sigma level.

## Conclusion

The research results show that the sense of stigma among middle-aged and young stroke patients is at a moderate or higher level. Currently, solutions for stroke patients’ feelings of stigma have yielded positive results [46, 47]. This study suggests that the independent influencing factors of stigma include age, pre-stroke monthly income, activities of daily living, positive emotions, and negative emotions. Therefore, formulating intervention strategies based on these influencing factors is particularly important to prevent the occurrence and development of stigma.

In conclusion, this study not only lays the foundation for future interventional studies on stigma in young and middle-aged stroke patients but also provides a reference for tertiary prevention of stroke patients to facilitate their early return to their families and society and to reduce the economic burden on families and society.

## Electronic supplementary material

Below is the link to the electronic supplementary material.

Supplementary Material 1

## Data Availability

All data generated or analyzed during this study are included in this published article [and its supplementary information files: Additional File 1].

## Abbreviations

SSS: Stroke Stigma Scale
PANAS: Positive and Negative Affect Scale
BI: Barthel Index
